# Lymphocytic interstitial pneumonia presenting with a ground glass nodule: A case report and literature review

**DOI:** 10.1097/MD.0000000000033613

**Published:** 2023-05-05

**Authors:** Qi Liu, Zhen Chen, Peng Deng, Jing Wang, Shengchu Zhang, Lihua Tang, Yuxia Yang, Bojuan Lang

**Affiliations:** a The First College of Clinical Medical Science, China Three Gorges University, Hubei, China; b Department of Cardiothoracic Surgery, Yichang Central People’s Hospital, Hubei, China; c Department of Breast Surgery, Yichang Central People’s Hospital, Hubei, China; d Department of Pathology, Yichang Central People’s Hospital, Hubei, China.

**Keywords:** case report, ground glass nodule, literature review, lymphocytic interstitial pneumonia

## Abstract

Lymphocytic interstitial pneumonia (LIP) is a rare benign lymphoproliferative disorder, often associated with autoimmune diseases. Most LIPs present with multiple bronchial cysts and diffuse interstitial infiltration. It is histologically characterized by widespread diffuse lymphocytic infiltration of the pulmonary interstitium, and the enlargement and widening of the alveolar septum.

**Patient concerns:**

A 49-year-old woman was admitted to hospital for finding pulmonary nodules for more than 2 months. 3D imaging chest computed tomography (CT) examination of both lungs showed that there was a middle lobe of the right lung with a size of about 1.5 cm × 1.1 cm ground-glass nodules.

**Diagnoses:**

A single operating port thoracoscopic wedge resection biopsy of a right middle lung nodule was performed. The pathology showed diffuse lymphocytic infiltration with varying numbers of small lymphocytes, plasma cells, macrophages and histiocytes infiltrating the alveolar septa, widened and enlarged alveolar septa, and scattered lymphoid follicles. Immunohistochemically, CD20 positive in follicular area, CD3 positive in interfollicular area. LIP was considered.

**Interventions:**

The patient was regularly followed without any specific treatment.

**Outcomes:**

Follow-up chest CT showed no significant abnormalities in the lungs 6 months after surgery.

**Lessons:**

To the best of our knowledge, our case may be the second reported case of a patient with LIP presenting with a ground glass nodule on chest CT, and it is speculated that the ground glass nodule may be an early manifestation of idiopathic LIP.

## 1. Introduction

Lymphocytic interstitial pneumonia (LIP), first reported by Carrington and Liebow^[1]^ in 1966, is an inflammatory lung reaction involving bronchial-associated lymphoid tissue that ultimately leads to reactive proliferation of T and B lymphocytes, plasma cells, and histiocytes and interstitial infiltration of the lung.^[2]^ Its incidence is low and most cases are secondary, especially in Sjogren’s syndrome.^[2]^ Patients with LIP lack specific clinical manifestations and imaging manifestations, and the final diagnosis depends on lung biopsy, which is easily misdiagnosed or missed in clinical diagnosis.^[3]^ By analyzing the clinical data of a patient with LIP who presented with ground glass nodules on chest computed tomography (CT) admitted to our hospital, and reviewing the relevant literature, we aim to further improve the understanding of LIP.

## 2. Case report

**Figure 1.**
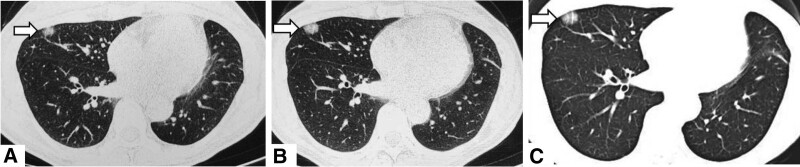
(A) Chest CT showed: the right lung middle lobe lateral segment subpleural see about 7.0 mm in diameter in the right middle lobe circular ground glass nodules, the edge of the lesion is unclear. (B) After 2 months, chest CT showed: subpleural section of lateral segment of middle lobe of right lung 10.0 × 13.0 mm-like round ground glass nodule. The lesion was slightly more dense and slightly enlarged in size compared with the previous one. (C) 3D imaging CT of both lungs showed: increased texture of both lungs, a mixed ground glass nodule shadow is seen in the outer segment of the middle lobe of the right lung, about 1.5 cm in size × 1.1 cm. CT = computed tomography.

**Figure 2.**
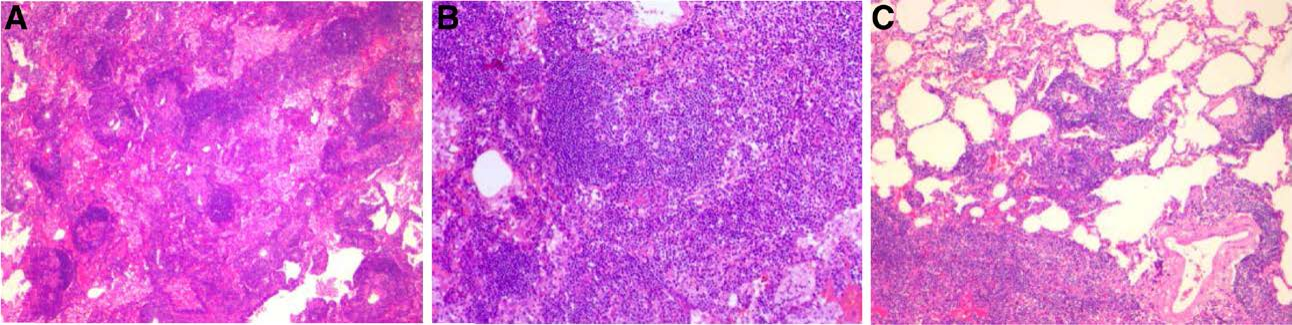
Lung, wedge biopsy. (A) Low magnification shows scattered distribution of lymphatic follicles (HE stain, original magnification, ×10). (B) The lung tissue shows a large number of lymphocytes and plasma cells infiltration, alveolar septum widening, visible lymphoid follicle formation, alveolar space visible plasma and many phagocytes aggregation (HE stain, original magnification, ×40). (C) The lesion site is not well defined with the surrounding alveoli, and the alveolar airspaces display variable degrees of distortion and collapse (HE stain, original magnification, ×40).

**Figure 3.**
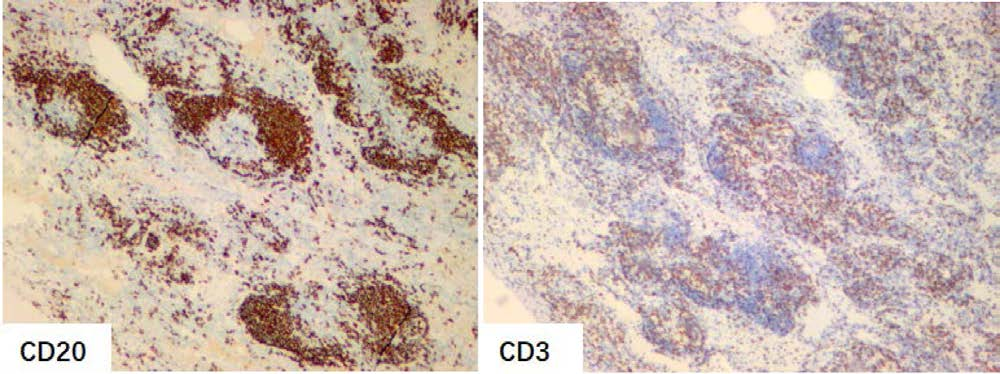
Photomicrographs of immunostained lung tissue. The follicular area was CD20 positive and alveolar septal lymphocytes were CD3 positive (original magnification, ×40).

A 49-year-old woman was admitted to the hospital with the chief complaint of “pulmonary nodules found for more than 2 months.” The patient reported that she went to the local hospital 2 months ago with “cough and sputum for 2 weeks” and that her chest CT showed “a ground glass nodule in the middle lobe of the right lung, about 0.7 cm in size” (Fig. 1A). The symptoms did not improve significantly after anti-infective treatment was given, and the chest CT was repeated (Fig. 1B). The patient was previously in good health and no pulmonary nodules were detected on chest CT last year. No positive signs were found on examination after admission to our hospital. Laboratory tests: negative for hepatitis B surface antigen, hepatitis C antibody, HIV and syphilis-specific antibodies; normal blood, urine and stool routine, liver function, coagulation and blood biochemistry; normal PCT, CRP and ESR; normal tumor marker set. The patient’s electrocardiogram, cardiac ultrasound and cranial CT showed no significant abnormalities. The lung function was normal. A 3D imaging CT examination of both lungs showed a ground glass nodule in the middle lobe of the right lung, measuring approximately 1.5 cm × 1.1 cm (Fig. 1C). Malignancy was not ruled out in this patient, and to clarify the nature of the nodule, a single operating port thoracoscopic wedge resection biopsy of a right middle lung nodule was performed under general anesthesia. The pathology showed diffuse lymphocytic infiltration with varying numbers of small lymphocytes, plasma cells, macrophages and histiocytes infiltrating the alveolar septa, widened and enlarged alveolar septa, and scattered lymphoid follicles (Fig. 2). Immunohistochemically, CD20 positive in follicular area, CD3 positive in interfollicular area (Fig. 3), Ki-67 showed high proliferation index in the germinal center, Bcl-6 and CD10 positive in the germinal center, CD38, IgG, IgG4, kappa, lambda scattered positive, CD21 positive in dendritic cells. LIP was considered.

Since LIP is mostly associated with immunodeficiency diseases, in order to exclude the presence of related primary diseases, rheumatoid immune-related tests were examined: MPO-ANCA: 0.22 mL/L, PR3-ANCA: <0.5 mL/L; rheumatoid factor antibodies: lgM: 0.1 U/mL, lgA: 0.1 U/mL, lgG: 0.1 U/mL; anti-cyclic citrullinated peptide antibody (anti-CCP antibody) < 0.50 U/mL; ENA antibody screening: except for anti-SSA/Ro52 antibody suggesting weak positivity, anti-dsDNA antibody, anti-nuclear chromatin antibody, anti-RNA antibody, anti-Scl-70 antibody and anti-Jo-1 antibody were all negative. The results of the review 2 months later were generally consistent with these results and did not suggest any significant abnormalities. At present, there are no examination results or clinical symptoms that can clearly suggest that the patient is suffering from Sjogren’s syndrome or other autoimmune diseases, and it is impossible to determine whether the patient’s LIP is secondary to Sjogren’s syndrome. However, in view of the patient’s weakly positive SSA antibodies, it is recommended to review the chest CT, rheumatologic immunity-related test results and serum protein regularly, and follow-up will be continued later. The patient is now in good general condition, with a cough that is more relieved than before. It is presumed that his cough symptoms may be related to the stimulation of inflammatory cells or immune cell infiltration within the small pulmonary nodules. Although invasive surgery was performed to clarify the benignity and malignancy of the nodules, the thoracoscopic lung wedge resection was less invasive and the patient recovered well after surgery. No postoperative complications affecting normal life such as chest tightness and shortness of breath were found, and other treatments such as drugs were not considered for the time being.

## 3. Discussion

LIP is a rare benign lymphoproliferative disorder whose exact pathogenesis is unknown and is often associated with autoimmune diseases (Sjogren’s syndrome, rheumatoid arthritis, systemic lupus erythematosus), gammoproteinemia, infections (human immunodeficiency virus and E-B virus), and genetic susceptibility.^[2,3]^ In determining the diagnosis of LIP, the focus is on identifying the underlying disease (e.g., connective tissue disease, infection) and distinguishing LIP from malignant pulmonary lymphoproliferative disorders (e.g., lymphoma) as well as nonspecific interstitial pneumonia.^[4]^

LIP mostly has an insidious onset and lacks typical clinical manifestations. Our patient had intermittent cough as the prominent clinical manifestation, and after admission to the hospital, relevant ancillary examinations were completed, and physical examination, pulmonary function, blood routine, and laboratory results were basically normal. Most patients had respiratory symptoms at the time of diagnosis, often presenting with cough, sputum and dyspnea, and progressive dyspnea and dry cough are now considered to be their main clinical characteristic manifestations,^[4,5]^ while systemic symptoms such as fever, night sweats and weight loss are relatively uncommon.^[6]^ Pulmonary function tests usually show restrictive ventilatory impairment.

The presentation of chest radiographs in patients with LIP lacks specificity and is mostly seen in the lower lung region, which usually shows bilateral reticular or reticulonodular-like shadows.^[7]^ As in other diffuse interstitial lung diseases, lung high-resolution CT is the radiological method of choice to determine the lung manifestations of LIP, with multiple bronchial cysts and diffuse interstitial infiltrates as its characteristic manifestations,^[8]^ and studies have found ground-glass shadows lobular centers or small subpleural nodules, thickened bronchovascular bundles, thickened lobular septa and cysts as well.^[3,9,10]^ The formation of a thin-walled cystic lumen may be associated with progressive obstruction and dilatation of the airway by lymphocytes infiltrating around the fine bronchi.^[11]^ Misdiagnosis and missed diagnoses can easily occur with imaging alone, and the combined use of several diagnostic methods can improve its diagnostic accuracy.^[12]^ The single ground glass nodule in the unilateral middle lobe of the lung demonstrated by chest CT in this patient was not a characteristic imaging presentation of LIP and needs to be differentiated from malignant tumors. Reviewing the previous literature on LIP, Kawaguchi et al^[13]^ reported the first case in which the CT presentation of LIP was similar to that of lung cancer. Seven months after thoracoscopic resection, a new nodule was found in this patient, and 2 years later, chest CT showed a hairy glass shadow, suggesting that the nodule-like lesion may be an early manifestation of LIP. Notably, neither this patient nor our patient had detectable underlying disease, and it is speculated that the ground glass nodule may be an early manifestation of idiopathic LIP. In this case, surgical excisional biopsy may not be the best diagnostic method. The nodule was >1 cm in diameter and close to the pleura, and a thoracentesis biopsy was feasible to determine the nature of the nodule.

The final diagnosis of LIP depends on lung biopsy, and current studies have shown diffuse lymphocytic infiltration of the interstitial lung (25%)^[8,14]^ with enlarged and widened lobular and alveolar septa^[3]^ as its main pathological manifestations. The infiltrating cells usually consist of lymphocytes, immunoblasts, plasma cells and histiocytes, including epithelioid cells and macrophages. Nodular lymphoid aggregates with reactive germinal centers are present in up to 50% of patients with LIP with idiopathic and connective tissue-related disease, in most cases with a greater proportion of lymphocytes compared to plasma cells,^[6,15]^ with B cells usually located in nodular lymphoid follicles and T cells mostly found in the interstitium.^[16]^ Compared to LIP, the interstitial lymphocytic infiltrate is less diffuse and intense in nonspecific interstitial pneumonia and hypersensitivity pneumonitis, and the lesions of nodular lymphoid hyperplasia are well-defined. Our patient’s postoperative pathology supports the diagnosis of LIP. Bronchoalveolar lavage is also a valuable indicator for the diagnosis of the disease, and bronchoalveolar lavage showing an increase in lymphocytes, CD3 cells and polyclonal CD20 cells generally suggests LIP,^[2,3]^ but the detection rate for clinical applications is not high and is usually used to identify or exclude underlying diseases such as lung infections.^[3]^

The systemic application of glucocorticoids is now considered the main treatment for LIP, and the principles of hormone administration need to be integrated with the combination of extrapulmonary autoimmune disease,^[3,6]^ and the use of other immunosuppressive agents, such as cyclophosphamide, chlorambucil and azathioprine, has varying effects.^[3]^ Long-term follow-up of LIP patients is sparse and individualized, but as a benign lymphoproliferative disease, most patients with LIP have a good prognosis.^[17]^ According to past studies, approximately 5% of patients with LIP convert to lymphoma later in life,^[8]^ but many researchers now also believe that those with LIP should have been diagnosed with malignant lymphoma at the outset, so the degree of risk of LIP converting to malignant lymphoma is not uniformly recognized, but there is still a need for long-term follow-up of diagnosed patients.^[18]^ Although it is now generally accepted that drug therapy such as glucocorticoids is recommended for interstitial lung disease with typical pulmonary imaging findings and impact on lung function, no cases have been reported for patients with solitary pulmonary nodule LIP. In this case, considering the adverse effects of glucocorticoids and the possibility of subsequent progression of the patient’s disease, we decided to perform conservative observation and regular follow-up.

## 4. Conclusion

Our case is highly rare and may be the second reported case of a patient with LIP presenting with a ground glass nodule on chest CT after Makoto Kawaguchi, and it is speculated that the ground glass nodule may be an early manifestation of idiopathic LIP.

## Acknowledgments

Thanks to my mom, my teachers, and my friends.

## Author contributions

**Formal analysis:** Zhen Chen, Peng Deng, Jing Wang.

Investigation: Qi Liu.

Resources: Peng Deng, Yuxia Yang, Bojuan Lang.

Supervision: Zhen Chen, Jing Wang.

Validation: Lihua Tang.

Writing – original draft: Qi Liu.

Writing – review & editing: Zhen Chen, Shengchu Zhang, Lihua Tang.
